# A perspective on precision medicine in obstructive sleep apnea: from pathophysiological phenotyping to integrated management pathways

**DOI:** 10.3389/fmed.2026.1776553

**Published:** 2026-04-13

**Authors:** Yan Chen, Si-Si Su, Ya-Ni Liu, Miao Han

**Affiliations:** 1 Department of Otolaryngology-Head and Neck Surgery, Affiliated Hospital of Yan'an University, Yan’an, China; 2 Department of Medicine, Yan'an Vocational and Technical College, Yan’an, China

**Keywords:** clinical phenotyping, obstructive sleep apnea, pathophysiological endotypes, personalized treatment, phenotype identification, precision medicine

## Abstract

Obstructive sleep apnea (OSA) is a condition defined by complex biological causes and highly varied symptoms. The traditional “one-size-fits-all” treatment approach, which depends on a single measurement like the Apnea-Hypopnea Index (AHI), frequently fails to provide individualized care, leading to poor results and low treatment adherence for many patients. Precision medicine offers a solution by using many different types of data to create detailed patient profiles. This data includes the specific physical causes of a patient’s OSA, their unique symptoms and other health conditions, biological markers, and information from ongoing monitoring. This detailed profiling allows doctors to group patients more precisely and match them to targeted treatments, such as procedures to open the airway, medications to stabilize breathing, and personalized lifestyle changes. The goal is to shift OSA care from a focus on standardized devices to plans built around the individual patient. To make this future a reality, the medical field needs standardized tools for patient profiling, strong clinical trials to prove these new methods work, and better teamwork across medical specialties. This coordinated effort is essential to make care for OSA more effective, personalized, and accessible to all who need it.

## 1 Introduction

Obstructive sleep apnea (OSA) is a common disorder marked by repeated upper airway collapse during sleep. It is highly prevalent and linked to serious health risks, including cardiovascular disease, metabolic syndrome, neurocognitive impairment, and significant daytime dysfunction (1). Despite its significant impact, managing OSA in clinical practice presents major challenges. While diagnostic methods are standardized and continuous positive airway pressure (CPAP) is the established first-line treatment, real-world effectiveness varies greatly (2). Many patients experience suboptimal responses to CPAP, often due to poor tolerance and low long-term adherence (2–4). Furthermore, the effectiveness of alternative treatments also differs substantially from person to person (2, 4). This variability demonstrates the limitation of a uniform approach, leaving a portion of patients without optimal management and highlighting the urgent need for personalized strategies based on a deeper understanding of the disease’s heterogeneity (3, 5).

Precision medicine is an emerging paradigm transforming chronic disease management. Its core principle shifts focus from general guidelines based on population averages to clinical decisions informed by an individual’s unique biological traits, physiological parameters, environmental exposures, and behavioral patterns (6). For OSA, this means moving beyond relying solely on the apnea-hypopnea index (AHI) as a universal severity metric toward defining distinct disease phenotypes (7, 8). These phenotypes represent subgroups driven by different underlying mechanisms, such as individual differences in upper airway anatomy, instability in respiratory control (high loop gain), arousal thresholds, and obesity-related physiological burden (9, 10). Integrating multi-dimensional data—including genomics, craniofacial imaging, upper airway physiology, sleep electroencephalogram features, symptom profiles, and co-existing conditions—enables more refined patient stratification (8, 11).

This perspective article aims to outline the application of precision medicine in OSA. We first explore advances in identifying OSA phenotypes based on pathophysiological mechanisms. Next, we analyze how to integrate this phenotypic information with clinical features to develop individualized treatment pathways. Our ultimate goal is to present a logical framework that connects precise diagnosis with integrated treatment. This framework seeks to advance OSA management from a generalized, device-centric model to a patient-tailored, precision medicine approach, potentially leading to more effective and targeted patient care.

Although this article is presented as a perspective rather than a systematic review, the conceptual framework is informed by a structured narrative literature assessment. Relevant publications were identified through searches of PubMed and Web of Science, with a focus on studies addressing OSA phenotyping, pathophysiological endotypes, precision medicine frameworks, and individualized therapeutic strategies. Priority was given to recent reviews, clinical trials, mechanistic investigations, and consensus statements published in peer-reviewed journals. Study selection was guided by relevance to multidimensional characterization of OSA and contribution to understanding phenotype-guided management approaches. This approach was adopted to ensure conceptual comprehensiveness while preserving the interpretative nature of a perspective article.

## 2 Heterogeneity of OSA: theoretical basis of precision medicine

### 2.1 Diversity in pathophysiological mechanisms

The rationale for applying precision medicine to OSA lies in the significant heterogeneity of the disorder. This heterogeneity stems from OSA not being a single disease, but rather a condition arising from different combinations of underlying biological mechanisms (12). Research has identified at least four primary contributing factors, known as endotypes: anatomical compromise of the upper airway, instability in respiratory control (high loop gain), a low arousal threshold, and poor responsiveness of the upper airway muscles (13). Anatomical compromise refers to physical narrowing that predisposes the airway to collapse. Respiratory control instability causes an overly sensitive breathing drive. A low arousal threshold means a patient wakes up too easily from sleep, and poor muscle responsiveness indicates the throat muscles fail to keep the airway open. The unique mix of these endotypes varies from person to person, creating distinct disease profiles (7). For example, one patient’s OSA might be caused mainly by a narrow airway, while another’s might stem from unstable breathing control and easy arousals, despite normal anatomy (13, 14). This diversity in causes explains why standard treatments like CPAP do not work equally well for everyone and provides the basis for developing therapies targeting a patient’s specific mechanisms (7).

### 2.2 Variability in clinical presentation and comorbidity profiles

OSA’s variability is also evident in how patients experience the disease and what other health problems they develop. Patients show clear differences in their main symptoms and severity (15, 16). A common presentation is significant excessive daytime sleepiness, which heavily impacts daily function (17). However, other patients may suffer mainly from broken sleep, frequent nighttime awakenings, or unrefreshing sleep, with little daytime sleepiness. The risks for related health conditions are not uniform either (15, 16). While OSA increases the overall risk for heart and metabolic diseases, this risk is not the same for all patients (17). Some patients have a stronger tendency to develop conditions like hard-to-control high blood pressure, heart rhythm disorders, or insulin resistance (18). Others may be more prone to problems with thinking skills or mood disorders like depression (19). These observations indicate that OSA encompasses multiple clinically relevant phenotypes characterized by distinct symptoms and health-risk trajectories.

Importantly, comorbid conditions play a central role in shaping personalized management strategies for OSA (11). Accumulating evidence indicates that cardiometabolic diseases, chronic inflammatory disorders, and neuropsychiatric conditions can modify disease expression, treatment responsiveness, and long-term prognosis (11, 20). Rather than representing merely secondary consequences, comorbidities may define clinically meaningful subgroups that warrant tailored therapeutic priorities (21). For instance, patients with predominant cardiovascular or metabolic comorbidity profiles may benefit from integrated risk-reduction strategies that combine respiratory therapy with systemic disease management (11). Recent analyses further suggest that incorporating comorbidity burden into phenotypic classification enhances clinical decision-making and supports a more holistic precision medicine framework in sleep apnea management (22).

Consequently, accurate identification of phenotype-specific clinical and comorbidity profiles is essential for predicting disease progression, optimizing therapeutic selection, and advancing individualized management strategies in patients with OSA (15, 16).

### 2.3 Limitations of traditional single-parameter diagnosis

For decades, the primary metric for diagnosing and categorizing OSA severity has been the AHI (7, 8). However, relying on the AHI alone has major limitations because it is a one-dimensional measure. First, the AHI does not reveal the underlying cause(s) of a patient’s OSA. Two patients with the same AHI score can have the disease for completely different reasons (11). Second, the AHI often correlates poorly with symptom burden. Someone with a high AHI might have few symptoms, while someone with a lower AHI might be severely impaired (2, 11). Finally, the AHI is a limited predictor for specific health complications. Other measures, like the severity and duration of low oxygen levels during sleep, may be better at forecasting heart-related risks (7, 23). Therefore, using the AHI as the sole guide for diagnosis and treatment is an oversimplification that can result in mismanagement. This highlights the need for a more comprehensive assessment that integrates multiple factors, such as detailed oxygen data, measures of sleep fragmentation, and specific symptom profiles (11, 23–25). The ultimate goal is to move toward a classification system based on underlying mechanisms (endotypes) and observable characteristics (phenotypes) to guide truly personalized therapy (7, 11).

## 3 A multidimensional strategy for OSA phenotype identification

**Figure 1:**
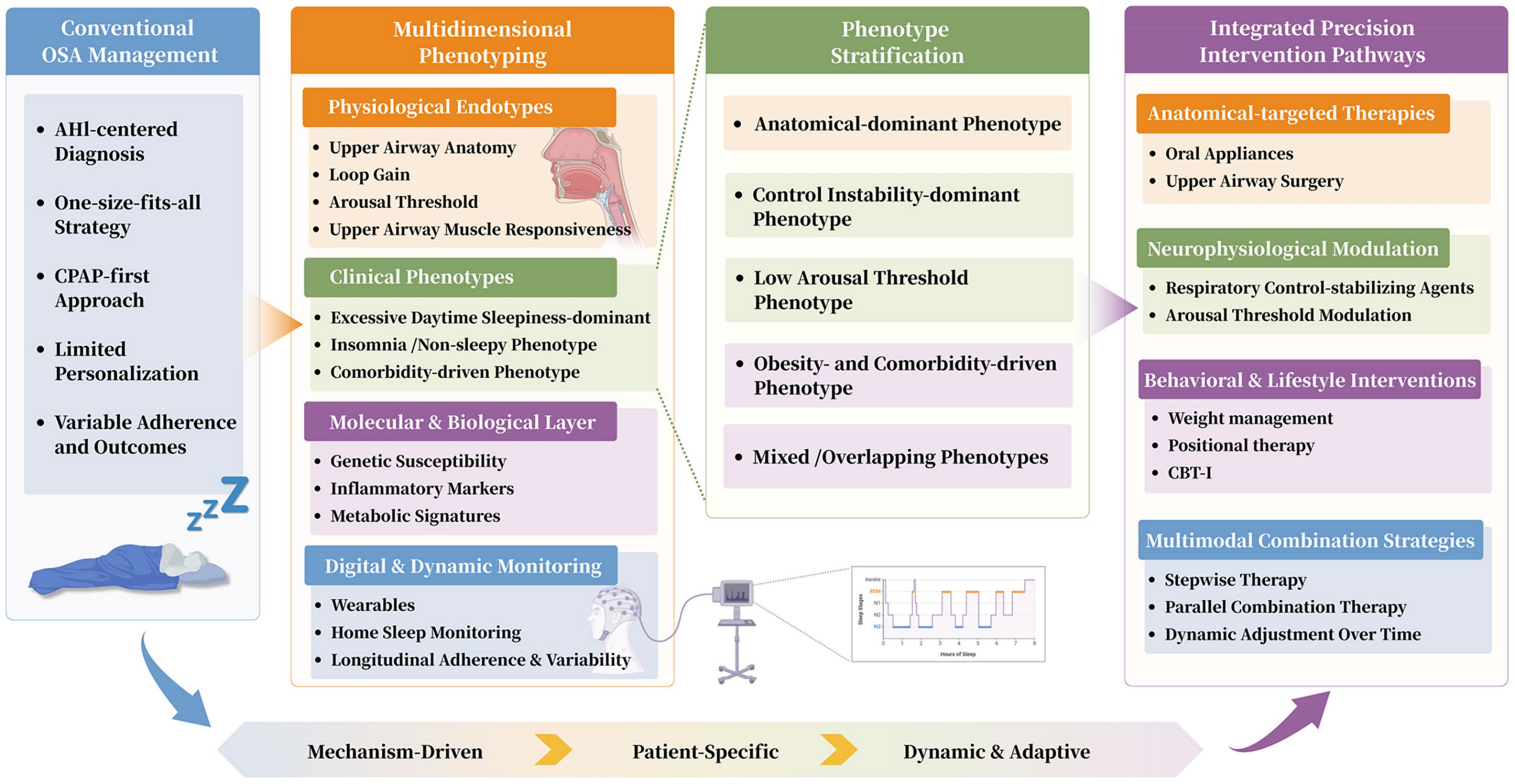
Precision medicine framework for obstructive sleep apnea: from multidimensional phenotyping to integrated treatment pathways.

Accurately identifying phenotypes in OSA is essential for achieving precision medicine (26). A single-dimension evaluation cannot fully capture the complexity of this disorder. Therefore, a multidimensional framework is required, integrating data from various sources and levels to identify phenotypes. This framework includes four core dimensions: physiological, clinical presentation, molecular biology, and dynamic behavioral monitoring. Together, these dimensions provide the foundation for developing personalized treatment plans (Figure 1; Supplementary Table 1).

### 3.1 Physiological phenotypes: quantifying endotypes based on mechanisms

The goal of physiological phenotyping is to identify an individual’s “endotype” by quantifying the specific mechanisms driving their OSA (27). Systematic methods, such as the PALM model, are designed to measure four key mechanisms: P (pharyngeal anatomy), A (arousal threshold), L (respiratory control stability), and M (upper airway muscle responsiveness) (27, 28). For example, loop gain can be calculated by applying mild pressure pulses during a sleep study. Similarly, the arousal threshold can be estimated by analyzing brain wave patterns (29). This mechanistic classification directly links a patient’s physiology to potential targeted treatments (30, 31). A patient with OSA caused mainly by unstable respiratory control might respond well to stabilizing medication, while a patient with a primary anatomical cause might be a better candidate for a dental appliance or surgery (30).

### 3.2 Clinical and symptom phenotypes: from patient-reported complaints to integrated risk stratification

Clinical phenotyping focuses on a patient’s symptoms and co-existing conditions, connecting biological mechanisms to daily life impact (8). Based on daytime symptoms, patients are often grouped into two main subtypes: “excessive daytime sleepiness-dominant” and “non-sleepy” (e.g., insomnia-dominant) (32). Another important category is the “comorbidity-driven” phenotype, which includes patients whose primary health risks are specific conditions like hypertension or metabolic syndrome linked to their OSA (16). Identifying these clinical profiles helps clinicians prioritize treatment goals and predict therapy tolerance and benefit (7).

### 3.3 Molecular and genetic phenotypes: exploring biomarkers and intrinsic susceptibility

Molecular phenotyping aims to discover the biological basis of OSA by exploring genetic risk factors, inflammatory signals, and metabolic changes. Research has found genetic variations linked to airway structure and the body’s response to low oxygen (33, 34). Measuring inflammation markers in the blood can help identify patients at higher cardiovascular risk (35). Analysis of metabolic byproducts can reveal unique chemical patterns associated with the stress of intermittent hypoxia (36). These molecular traits are not just disease indicators but may also serve as tools to predict treatment response.

### 3.4 Digital health and real-time monitoring data: capturing dynamic and real-world phenotypes

Traditional sleep studies provide limited data from a single night in a lab. In contrast, digital tools like wearable devices and home monitors allow for continuous tracking in a patient’s daily life (37–39). These technologies can monitor sleep patterns, oxygen levels, and heart rate over long periods (37, 39). This reveals dynamic phenotypes, such as position-dependent or highly variable OSA. Analyzing this continuous data with advanced algorithms provides a more accurate picture of a patient’s real-world disease burden and treatment adherence, enabling more responsive and personalized management (40, 41).

## 4 From phenotype to precision intervention: constructing an integrated treatment pathway

**Figure 2:**
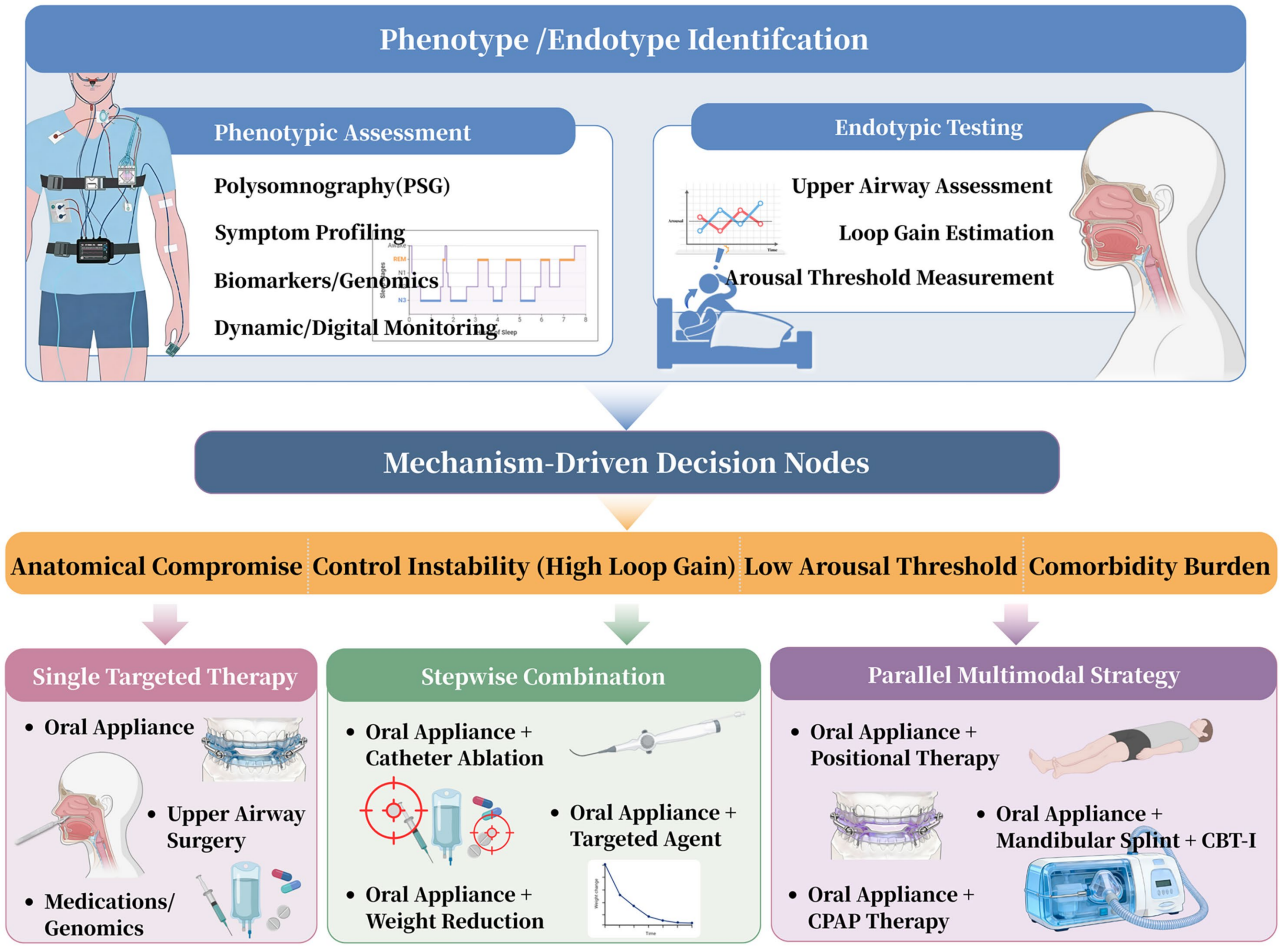
Translational pathway linking OSA phenotypes to targeted and multimodal interventions.

The primary objective of precision medicine for OSA is to translate a patient’s identified phenotype into a tailored intervention. An integrated treatment plan should replace the traditional trial-and-error method, with therapeutic strategies selected, combined, and refined based on a patient’s comprehensive phenotypic profile (Figure 2). Instead, strategies should be selected, combined, and refined systematically based on the patient’s comprehensive phenotypic profile. This plan focuses on four main areas of intervention: anatomical, neurophysiological, behavioral, and multimodal combination (Figure 1). The aim is to deliver a mechanism-specific treatment, thereby improving its effectiveness and the patient’s long-term commitment.

Importantly, the strength of evidence supporting these phenotype-guided interventions varies considerably. Established therapies—including CPAP optimization, oral appliance therapy, weight reduction, and positional therapy—are backed by large clinical studies and guideline recommendations (42–44). In contrast, several pharmacological strategies, neuromodulatory interventions, and approaches based on intermittent hypoxia remain investigational, currently supported primarily by mechanistic studies or proof-of-concept clinical trials (45, 46). Accordingly, precision medicine in OSA should be understood as an evolving translational framework, in which therapeutic adoption should remain aligned with the strength of available clinical evidence.

### 4.1 Targeted therapies based on anatomical abnormalities

For patients whose OSA is mainly caused by physical airway blockage, treatment should focus on enlarging or stabilizing the airway. Upper airway surgery should be considered only after detailed imaging confirms a specific, surgically correctable obstruction (47). Oral appliances, which reposition the jaw forward, work best for patients with certain anatomical features like a receding jaw or large tongue (48). Choosing the right treatment requires precise anatomical assessment to avoid ineffective procedures for patients whose OSA stems from non-anatomical causes (49). A phenotype-guided approach allows for the customization of device settings and predicts which patients will tolerate and benefit from long-term use.

### 4.2 Interventions based on neuromodulation and respiratory stability

Patients whose OSA is driven by unstable breathing control or who wake up too easily require treatments that target these neurological factors (13, 50). The drug acetazolamide can help stabilize irregular breathing patterns, especially in patients with this specific physiological trait (51). Certain medications, like the antidepressant oxybutynin, may help patients sleep more soundly by reducing sensitivity to minor breathing disruptions (52, 53). Furthermore, carefully supervised intermittent hypoxia training might help the body better tolerate brief drops in oxygen during sleep, potentially lowering related stress on the heart (54). The success of these treatments depends on accurately identifying the patients who have the specific physiological mechanisms these interventions are designed to correct.

It should be emphasized that several pharmacological and neuromodulatory approaches discussed above remain under active investigation (55, 56). Although early studies demonstrate promising physiological effects, many interventions are currently supported primarily by small-scale trials or proof-of-concept studies (57, 58). These strategies should therefore be interpreted as emerging therapeutic directions rather than established standards of care, underscoring the need for large randomized clinical trials to confirm their efficacy, safety, and long-term clinical benefit (57).

### 4.3 Individualized behavioral and lifestyle interventions

Behavioral strategies are essential but should be customized rather than one-size-fits-all. For OSA related to obesity, a structured and intensive weight management program is a critical part of treatment (59). The approach should be tailored to the individual’s specific metabolic profile and body fat distribution. Positional therapy, which prevents sleeping on one’s back, is specifically for patients whose breathing issues occur primarily in that position (60). For patients who struggle mainly with insomnia or anxiety, cognitive behavioral therapy for insomnia should be a primary focus to improve sleep habits and mental well-being (61). Personalizing behavioral plans in this way leads to better patient engagement and long-term success.

### 4.4 Designing multimodal combination treatment pathways

Since most patients have a mix of underlying causes, a single treatment is often not enough. Treatment plans should therefore be designed as either “stepped” (adding therapies sequentially) or “parallel” (starting multiple therapies at once) based on the combination of phenotypes (62–64). For example, a patient might start with an oral appliance for an anatomical issue and later add a medication if unstable breathing persists (65). A patient with both severe obesity and position-dependent OSA might begin weight management and positional therapy simultaneously (66). These pathways should be guided by evidence-based tools that incorporate ongoing data from sleep studies and patient feedback. The goal is to create a flexible, patient-centered treatment plan that adapts over time to deliver the best possible health outcomes.

## 5 Challenges and future directions

While a phenotype-based approach promises to transform how we treat OSA, it faces major hurdles before becoming a standard part of clinical care. These challenges include technical issues, practical difficulties in the clinic, and important ethical questions. Together, they define the complexity of this shift in medical practice. Identifying these specific problems and planning how to solve them is essential for moving this new approach from the research lab to the patient’s bedside.

### 5.1 Technical bottlenecks

The first major challenge is technological. There is no agreed-upon, standard way to measure the core physiological traits that cause OSA (7, 10). Methods differ between labs, making it hard to compare results or use these tools widely in clinics (10). Furthermore, we have advanced tools that can generate huge amounts of biological data (11). However, it remains difficult to combine this complex molecular information with a patient’s symptoms and sleep study results to make clear treatment decisions (11). Creating standard, user-friendly tools for this purpose is a critical first step.

### 5.2 Barriers to clinical implementation

Putting these new ideas into practice in everyday clinics presents another set of problems. Specialized equipment and expert analysis are often not available outside of major medical centers (7, 67). This creates an access problem for patients in smaller communities or with fewer resources (67). We also need to prove that this detailed, personalized approach is worth the extra cost (11). Studies should show it leads to better long-term health and is cost-effective compared to standard care (11). Finally, doctors and other healthcare providers need training to understand and use these new concepts and tools effectively (67).

### 5.3 Future research directions

To overcome these obstacles, future research should focus on three key areas. First, large, well-designed clinical trials are needed to prove that treatment plans based on a patient’s phenotype lead to better results (30). Second, we should develop smart computer systems that can analyze all the different types of patient data and help doctors choose the best treatment (68). Third, we should find ways to deliver this personalized care using telemedicine and digital health apps to reach more patients efficiently (69).

### 5.4 Ethical and societal considerations

Alongside technical progress, we should carefully consider ethical and social issues. Protecting patient data is extremely important, especially when dealing with sensitive genetic and health information (70, 71). We should also ensure fair access to these advanced treatments so they do not worsen existing health inequalities (67). Finally, patients need to be well-informed partners in their care, understanding their own health data and the reasons behind their treatment options (72).

## 6 Summary

Precision medicine is changing how we approach OSA. It aims to replace the traditional one-size-fits-all model with a personalized, integrated strategy. This change is possible because we now better understand that OSA is a highly variable disease. This variability exists in its underlying causes, how it appears in patients, and how patients respond to treatment. By identifying specific patient subgroups (phenotypes), doctors can look beyond just the AHI number. They can instead tailor both diagnosis and treatment to the individual’s unique profile. The ultimate goal is to improve treatment success, help patients stick with their therapy, and achieve better overall health outcomes by treating the root causes of the disorder.

Making this new approach a reality requires action on several fronts. First, it demands strong teamwork across different medical and scientific specialties. Second, we urgently need new clinical studies that test whether treatments chosen for a specific phenotype work better than standard care. Finally, we should integrate the successful strategies from this research into everyday medical practice and guidelines. By focusing on these steps, the medical community can move toward a future of more precise, effective, and fair care for all patients with OSA.

## Data Availability

The original contributions presented in the study are included in the article/Supplementary material, further inquiries can be directed to the corresponding author.
