# Supplementary material for: A perspective on precision medicine in obstructive sleep apnea: from pathophysiological phenotyping to integrated management pathways
Source: Front Med (Lausanne). 2026 Apr 13;13:1776553. doi: 10.3389/fmed.2026.1776553 (PMC13116003; doi:10.3389/fmed.2026.1776553)
Supplement: Supplementary file 1 [file Table_1.DOCX]

**Table 1. Representative OSA phenotypes, key characteristics, assessment tools, and precision treatment strategies**

| **OSA phenotype** | **Dominant mechanisms** | **Key clinical features** | **Representative assessments** | **Precision intervention** |
| --- | --- | --- | --- | --- |
| Anatomical-  dominant | Upper airway collapse | Loud snoring, positional OSA | Imaging, cephalometry | Oral appliance, surgery |
| High loop gain | Ventilatory instability | Central tendency, hypoxia | polysomnography-  derived loop gain | Acetazolamide |
| Low arousal threshold | Frequent awakenings | Insomnia-  dominant | EEG-based metrics | Sedative modulation, CBT-I |
| Comorbidity-  driven | Metabolic/CV burden | Hypertension, diabetes | Biomarkers, risk profiling | Multimodal, risk-focused |

**Abbreviations**: OSA, obstructive sleep apnea; CBT-I, cognitive behavioral therapy for insomnia; EEG, electroencephalography; CV, cardiovascular.
